# Kind – und dann? Wandel partnerschaftlicher Erwerbsverläufe drei Jahre nach dem Übergang in die Elternschaft

**DOI:** 10.1007/s11577-022-00860-5

**Published:** 2022-10-21

**Authors:** Nadiya Kelle, Laura Romeu Gordo, Julia Simonson

**Affiliations:** Deutsches Zentrum für Altersfragen (DZA), Berlin, Deutschland

**Keywords:** Geburt des ersten Kindes, Elternpaare, Erwerbsarrangements, Kohortenvergleich, Sequenzmusteranalyse, First childbirth, Parental couples, Employment arrangements, Cohort Comparison, Sequence analysis

## Abstract

Der Übergang in die Elternschaft markiert für viele Elternpaare den Übergang in geschlechterspezifische Erwerbsarrangements, oft unabhängig von der gelebten vorgeburtlichen Arbeitsteilung. Dabei können die Entscheidungen über die Erwerbsarrangements nach der Geburt des ersten Kindes richtungsgebend für die zukünftigen Erwerbsverläufe und Alterssicherung sein. Vor diesem Hintergrund fokussiert der Beitrag auf zwei Fragen: erstens, ob sich gerade für jüngere Elternpaare der in den 1980er-Jahren Geborenen eine Konvergenz in den Erwerbsverläufen nach dem Übergang in die Elternschaft im Vergleich zu den in den 1970er-Jahren geborenen Elternpaaren zeigt und zweitens, ob die Arbeitsteilung vor dem Übergang in die Elternschaft eine zunehmende Rolle für die Erwerbskonstellationen danach spielt. Unter Verwendung der Daten des Sozio-oekonomischen Panels (SOEP) werden anhand sequenz-, cluster- und regressionsanalytischer Verfahren Erwerbsverläufe von 900 Paaren über 36 Monate nach dem Übergang in die Elternschaft analysiert. Für die Kohorte der in den 1980er-Jahren Geborenen setzt sich die Bedeutungsabnahme traditioneller Erwerbsarrangements fort. Zudem gibt es immer mehr Elternpaare, in denen beide Elternteile relativ schnell nach der Geburt eines Kindes in die Erwerbstätigkeit zurückkehren. Kaum zu beobachten ist, dass Väter ihre Erwerbstätigkeit zunehmend zugunsten einer stärkeren Einbindung in Haushalts- oder Familientätigkeiten einschränken. Hingegen scheinen die Konvergenzen in den Erwerbsverläufen zwischen Müttern und Vätern vielmehr ein Resultat zunehmender Erwerbsdiskontinuitäten zu sein. Darüber hinaus hat die vorgeburtliche Arbeitsteilung auch für die jüngeren Elternpaare einen eher geringen Einfluss auf ihre nachgeburtlichen Erwerbskonstellationen. Die Ergebnisse legen nahe, dass der Abbau von geschlechterspezifischen Ungleichheiten am Arbeitsmarkt verstärkt voranzutreiben ist, damit weitere Anreize für die gleichmäßigere Erwerbsaufteilung im Paarkontext entstehen können.

**Zusatzmaterial online:**

Zusätzliche Informationen sind in der Online-Version dieses Artikels (10.1007/s11577-022-00860-5) enthalten.

## Abstract (en)

For many couples, the transition to parenthood also marks a transition to gender-specific employment arrangements. However, decisions about employment arrangements after the birth of the first child can be critical for future employment patterns and old-age provision. This article focuses on two questions: first, whether after the transition to parenthood there is a convergence in the employment patterns of younger parental couples born in the 1980s compared with birth cohorts born in the 1970s, and second, whether a division of labour before the transition to parenthood plays an increasing role in employment arrangements afterwards. Using data from the German Socio-Economic Panel (SOEP), we analysed the employment patterns of 900 couples over 36 months after the transition to parenthood using sequence, cluster, and regression analysis methods. It was shown that even though traditional employment arrangements have declined in importance, they continue to dominate. Furthermore, the convergence observed is due more to the increasing discontinuities in men’s employment trajectories than to the birth of the child. Moreover, the prebirth division of labour has a rather minor influence on the postbirth employment arrangements of younger parental couples. The findings suggest that greater efforts should be made to reduce gender inequalities in the labour market in order to create further incentives for a more equal division of labour in the couple context.

## Einleitung

Der Vater ist erwerbstätig, die Mutter kümmert sich um den Haushalt und die Kinder – das Alleinverdienermodell der Nachkriegszeit prägte Jahrzehnte lang die familiäre Arbeitsteilung in Westdeutschland. Seitdem hat sich einiges verändert: Die Forschung der letzten Jahre zeigt, dass Mütter nach der Geburt eines Kindes immer schneller in das Erwerbsleben zurückkehren (Frodermann et al. 2013; Ziefle und Gangl 2014) und dass Väter gerade in den ersten Lebensjahren des Kindes zu größeren Anteilen in die Kinderbetreuung involviert sind (Bünning 2016; Schober und Zoch 2019; Tamm 2019). Zugleich ist festzustellen, dass der Übergang in die Elternschaft weiterhin oft mit einer Stärkung der geschlechterspezifischen Arbeitsteilung verbunden ist (Grunow et al. 2007, Dechant et al. 2014). So geben Frauen ihre Erwerbstätigkeit in dieser Zeit oftmals (zeitweise) auf oder reduzieren ihre Arbeitsstunden, während Väter in vielen Fällen weiterhin (vollzeit-)erwerbstätig sind (Pollmann-Schult 2015; Kühhirt 2012). Während Mütter häufig eine längere Elternzeit nehmen, gehen viele Väter über zwei „Vätermonate“ nicht hinaus (Bünning 2016; Samtleben et al. 2019).

Die traditionellen Erwerbsarrangements in den ersten Monaten und Jahren nach dem Übergang in die Elternschaft können weit in die Zukunft reichende Konsequenzen für die nachfolgenden Erwerbsverläufe und für die Verfestigung von Geschlechterungleichheiten auf dem Arbeitsmarkt haben. Für Frauen zeigen sich negative Konsequenzen in Form von geringeren Chancen auf berufliche Weiterbildungen und Leitungspositionen sowie geringeren Stundenlöhnen (Adda et al. 2017; Gallego Granados und Wrohlich 2018; Giesecke 2009). Vor allem längere Elternzeiten können für Frauen zu Lohnnachteilen führen (Lott und Eulgem 2019; Schmelzer et al. 2015) und im Zusammenhang mit niedrigeren Alterseinkommen stehen. So sind Frauen in Westdeutschland mit diskontinuierlichen und durch Kindererziehungszeiten geprägten Erwerbsverläufen besonders von Niedrigrenten betroffen (Brussig und Zink 2018). Auch generell gehen geschlechterspezifische Erwerbsarrangements mit einem geringeren individuellen Alterseinkommen von Frauen einher (Keck und Romeu Gordo 2020; Möhring und Weiland 2021).

Bisherige Forschungsarbeiten verdeutlichen demnach, dass die Gleichstellung zwischen Müttern und Vätern noch lange nicht erreicht ist und dass die geschlechterspezifischen Erwerbsarrangements nach dem Übergang in die Elternschaft negative Konsequenzen für die Erwerbsverläufe und Alterssicherung von Frauen haben können (z. B. Hobler et al. 2020; Steiber und Haas 2010). Jedoch bleibt bisher die Frage weitgehend unbeantwortet, ob im Vergleich unterschiedlicher Geburtskohorten Konvergenztendenzen zwischen Müttern und Vätern in den partnerschaftlichen Erwerbsverläufen die ersten Monate und Jahre nach dem Übergang in die Elternschaft zu beobachten sind. Der Beitrag adressiert diese Forschungslücke und fokussiert auf partnerschaftliche Erwerbskonstellationen im Kohortenvergleich nach dem Übergang in die Elternschaft. Es wird untersucht, inwiefern sich im Zeitverlauf Veränderungen in Richtung einer Konvergenz der Erwerbsverläufe von Frauen und Männern im Partnerschaftskontext ergeben haben.

Die Studie geht in wesentlichen Punkten über die bisherige empirisch fundierte Literatur hinaus. Erstens nutzt sie dyadische Daten von Paaren, mit denen sich die Prozesse der partnerschaftlichen Arbeitssteilung im Haushalt umfassend darstellen lassen. Zudem konzentriert sie sich zweitens nicht auf einzelne Aspekte (z. B. Wiedereinstieg von Müttern in die Erwerbstätigkeit, Aufnahme der Elternzeiten von Vätern), sondern betrachtet unter Verwendung von sequenz- und clusteranalytischen Verfahren die partnerschaftlichen Erwerbskonstellationen in den ersten drei Jahren nach der Geburt in Gänze. Dies erlaubt es, die Zeiten in Erwerbstätigkeit, Haushalts‑/Kinderbetreuungstätigkeit sowie sonstiger Nichterwerbstätigkeit und auch die Übergänge zwischen den einzelnen Erwerbsstatus nachzuvollziehen. Somit kann ein Gesamtbild über die Verläufe unmittelbar nach dem Übergang in die Elternschaft gewonnen werden. Drittens wird außerdem anhand eines Vergleichs von Kohorten geboren in den 1970er- und 1980er-Jahren untersucht, inwiefern sich ein Wandel bezüglich der partnerschaftlichen Erwerbsverläufe sowie ein Wandel im Einfluss der vorgeburtlichen Erwerbskonstellationen auf die partnerschaftlichen Erwerbskonstellationen in den ersten Jahren nach dem Übergang in die Elternschaft beobachten lässt. Die zentralen Forschungsfragen sind: Wie gestalten sich die partnerschaftlichen Erwerbsverläufe in den ersten Monaten nach dem Übergang in die Elternschaft? Inwiefern ist hierbei über die Kohorten hinweg eine Konvergenz der Erwerbsverläufe zwischen Frauen und Männern im Partnerschaftskontext zu beobachten? Und welche Rolle spielt die partnerschaftliche Arbeitsteilung vor der Erstgeburt auf die unmittelbare Ausgestaltung der Erwerbskonstellationen danach?

Für die Analysen werden Daten des Sozio-oekonomischen Panels (SOEP) verwendet. Die Datengrundlage erfüllt die hohen Voraussetzungen der Analysen. Das SOEP ist eine Langzeithaushaltsbefragung, sodass Informationen auf Paarebene bezogen werden können (Goebel et al. 2019). Darüber hinaus ermöglichen die vorliegenden Kalenderinformationen die Identifizierung monatsgenauer Angaben zum Zeitpunkt der Geburt eines Kindes sowie zum Erwerbsstatus beider Elternteile im Zeitvergleich. Zudem bieten die Daten einen langen Beobachtungszeitraum mit insgesamt 36 Erhebungswellen, sodass drei Jahre nach der Geburt des ersten Kindes beobachtet und verschiedene Geburtskohorten identifiziert und miteinander verglichen werden können.

Im Folgenden werden zunächst die strukturellen Rahmenbedingungen in Deutschland im Wandel dargestellt. Daraufhin wird die Frage, inwiefern ein Wandel nachgeburtlicher partnerschaftlicher Erwerbskonstellationen zu erwarten ist, aus theoretischer Perspektive beleuchtet und es werden Forschungshypothesen formuliert. Anschließend werden die verwendeten Daten und Methoden erläutert und die Ergebnisse der Sequenz- und Clusteranalyse sowie der multinomialen logistischen Regression dargestellt. Der Artikel schließt mit einer Diskussion und Schlussbetrachtung.

## Strukturelle Rahmenbedingungen im Kohortenvergleich

In den 2000er-Jahren fanden in der deutschen Familienpolitik bedeutsame Reformen statt. Der politische Hintergrund dieser Reformen war, Anreize für frühere (Wieder‑)Erwerbseinstiege für Mütter sowie längere Familienzeiten für Väter zu schaffen (vgl. Schutter und Zerle-Elsäßer 2012; Ziefle und Gangl 2014). So wurde mit den Gesetzen von 2005 und 2006 der Kitabetreuungsausbau vorangetrieben mit dem Ziel, mehr Betreuungsplätze für Kinder unter drei Jahren zu schaffen. Im Jahr 2013 wurde für Kinder ab dem vollendeten ersten Lebensjahr ein Rechtsanspruch auf einen Betreuungsplatz eingeführt. Tatsächlich ist die Betreuungsquote von Kindern unter drei Jahren von 13,6 % im Jahr 2006 auf 35,0 % im Jahr 2020 gestiegen (BMFSFJ 2020; Statistisches Bundesamt 2020).

Ferner wurde mit der Elterngeldreform aus dem Jahr 2007 die bisherige Erziehungszeit reformiert. Zwar gab es bereits in den 1990er-Jahren Erweiterungen der Erziehungszeit – diese wurde im Jahr 1990 von 15 auf 18 Monate und im Jahr 1992 auf 36 Monate ausgeweitet. Damit wurden jedoch weder Anreize für die Erwerbstätigkeit von Müttern noch Anreize für die Familientätigkeit von Vätern gesetzt. Dies änderte sich mit dem Gesetz zum Elterngeld und zur Elternzeit (BEEG) von 2007, das gezielt Anreize für die Beteiligung von Vätern an der Elternzeit sowie die schnellere Rückkehr von Müttern auf den Arbeitsmarkt setzte (Brandt 2017; Geisler und Kreyenfeld 2019;). Das Elterngeld wird im Gegensatz zum Erziehungsgeld einkommensabhängig bezahlt. Es können maximal zwölf Monate Elterngeld durch ein Elternteil beantragt werden. Wenn beide Elternteile Elterngeld beantragen, gibt es zwei zusätzliche Partnermonate (oftmals bezeichnet als „Vätermonate“). Somit beträgt die Elternzeit mit den zwei zusätzlichen Partnermonaten insgesamt maximal 14 Monate. Diese können abgesehen von den zwei Partnermonaten frei zwischen den Eltern aufgeteilt werden. Tatsächlich ist die Väterbeteiligung am Erziehungs- bzw. Elterngeldbezug deutlich gestiegen: 2006 lag sie noch bei 3,0 %, 2019 bei bereits 42,1 % (BMFSFJ 2021). Im Jahr 2015 wurde ElterngeldPlus eingeführt und damit wurden Rahmenbedingungen für den Elterngeldbezug für Elternpaare geschaffen, die in Teilzeit erwerbstätig sind. Dabei wird die Bezugshöhe entsprechend angepasst und die Bezugsdauer (auch für die Partnermonate) verdoppelt sich (Huebener et al. 2016).

Zudem wurden in den 2000er-Jahren sozialpolitische Maßnahmen beschlossen. Dazu gehören Reformen zur Lockerung von Arbeitsmarktregelungen wie das Teilzeit- und Befristungsgesetz (TzBfG) von 2001 sowie die Hartz-Reformen (Hartz I–IV) der 2000er-Jahre. Dabei wurden unter anderem Teilzeitarbeit und geringfügige Beschäftigung ausgeweitet. Infolgedessen wurde Erwerbstätigkeit in Teilzeit selbstverständlicher: Der Anteil Teilzeitbeschäftigter ist insgesamt von 19,5 % im Jahr 1999 auf 29,2 % im Jahr 2019 gestiegen (IAQ 2020a). Etwa 6–7 % entfallen dabei auf geringfügige Beschäftigung (IAQ 2020b). Die Erwerbstätigenquoten von Müttern von Kindern unter drei Jahren sind seit 1999 signifikant gestiegen, was insbesondere auf die gestiegene Teilzeitbeschäftigung zurückzuführen ist (IAQ 2020c). Auch befristete Beschäftigung ist in den letzten zwanzig Jahren leicht gestiegen, dies hat unter anderem zur Diskontinuität von Erwerbsverläufen beigetragen (WSI Gender Datenportal 2020; Hohendanner 2010).

Um zu untersuchen, inwiefern sich die partnerschaftlichen Konstellationen in den ersten Jahren nach dem Übergang in die Elternschaft gewandelt haben, werden drei Geburtskohorten miteinander verglichen: 1970–1974, 1975–1979 und 1980–1984. Es wird erwartet, dass sich die partnerschaftlichen Verläufe angesichts der politischen Interventionen insofern gewandelt haben, dass Frauen nach dem Übergang in die Elternschaft mehr Zeit in Erwerbstätigkeit verbringen und Männer mehr Zeit für Haushalts- und Familienarbeit aufwenden. Darüber hinaus wird erwartet, dass die Erwerbsverläufe in Verbindung mit politischen Maßnahmen zur Ausweitung von Teilzeit- und geringfügiger sowie befristeter Beschäftigung unbeständiger und diskontinuierlicher geworden sind. Für die Analyse wurden Kohorten ausgewählt, die in unterschiedlichem Umfang von diesen Maßnahmen betroffen sind. Zugleich haben die meisten Paare aus diesen Kohorten bereits ihr erstes Kind bekommen, was für spätere Geburtskohorten noch nicht der Fall ist. Außerdem liegen für die jüngeren Geburtskohorten im Gegensatz zu den älteren alle für die Analysen notwendigen Informationen (wie monatsgenaue Angaben zur Kindergeburt und zum Erwerbsstatus) vor.

**Figure 1:**
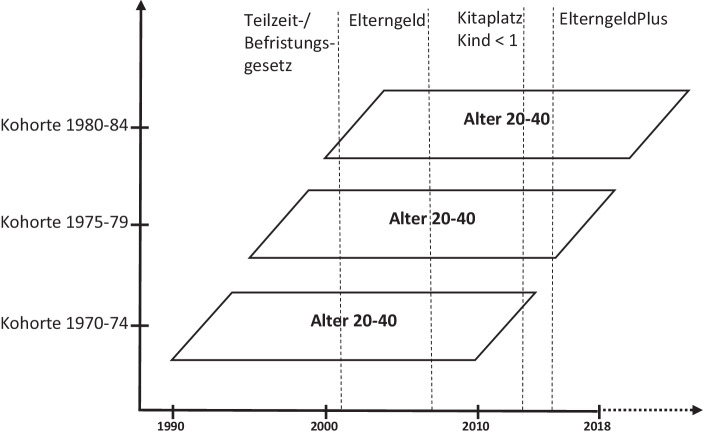

In Abb. 1 werden die drei Geburtskohorten über die historische Zeit hinweg dargestellt. Zu sehen sind die Kohorten im Alter zwischen 20 und 40 Jahren, also dem Alter, in dem zumeist das erste Kind geboren wird. Die Abbildung verdeutlicht, dass die betrachteten Kohorten zu unterschiedlichen Zeitpunkten in ihrem Lebensverlauf von den dargestellten Reformen betroffen sind.^1^ Es zeigt sich beispielsweise, dass die Elterngeldreform in den Beginn der Familiengründungsphase der Kohorte 1980–1984 fiel, für Kohorte 1970–1974 kam sie hingegen eher gegen Ende der Familiengründungsphase. Außerdem wurde die Kohorte 1970–1974 im Gegensatz zu den Kohorten 1975–1979 und 1980–1984 nicht von allen Reformen betroffen. Zugleich haben diese Reformen für die zwei jüngeren Kohorten zu unterschiedlichen Zeitpunkten im Lebensverlauf eine Rolle gespielt. Anzunehmen ist, dass sich die politischen Interventionen mit höherer Wahrscheinlichkeit in den partnerschaftlichen Erwerbsverläufen der jüngsten Kohorte widerspiegeln. Die sozialpolitischen Reformen ab Anfang der 2000er-Jahre sollten sich ebenfalls vor allem auf die partnerschaftlichen Erwerbsverläufe der jüngsten Kohorte auswirken.

## Wandel der Erwerbskonstellationen von Elternpaaren?

### Theoretischer Hintergrund und Stand der Forschung

Die traditionellen geschlechterspezifischen Erwerbsarrangements im Zuge der Elternschaft werden nach dem haushaltsökonomischen Ansatz von Becker (1993) durch eine bessere Humankapitalausstattung des Mannes und einen höheren gemeinsamen Nutzen für die Partnerin und den Partner begründet. Die höheren Humankapitalinvestitionen von Männern lassen sich der Theorie zufolge wiederum auf die (erwarteten) geschlechterspezifischen Lebensverläufe und die durch familienbedingte Erwerbsunterbrechungen (erwartete) geringere Rendite bei Frauen zurückführen. Der ressourcentheoretische Ansatz betont den individuellen Nutzen der innerhalb eines Paares ausgehandelten Arbeitsteilung (Blood und Wolfe 1960). Dabei wird unterstellt, dass Personen einen individuellen Nutzen eher in der Erwerbstätigkeit sehen, Haushaltstätigkeiten dagegen eher versuchen zu minimieren. Die Bargaining-Theorie berücksichtigt die Verhandlungsmacht in der Partnerschaft: Die Person mit einer besseren ökonomischen Ressourcenausstattung hat demnach höhere Chancen für die Erwerbstätigkeit (Manser und Brown, 1980). Tatsächlich beobachten Studien für Kohorten geboren in den 1960er-Jahren eine Abkehr vom klassischen Alleinverdienermodell, welche unter anderem auf bessere Einkommenspotenziale sowie eine immer bessere Humankapitalausstattung von Frauen zurückzuführen ist (Simonson et al. 2011; Trappe et al. 2015). Gleichzeitig deuten Studien auf eine weiterhin traditionelle Arbeitsteilung nach dem Übergang in die Elternschaft hin – trotz der Tatsache, dass vor der Geburt des ersten Kindes fast die Hälfte aller Frauen in Europa „Familienernährerinnen“ sind (Dieckhoff et al. 2020; Grunow 2016). Für Frauen ist die Elternschaft mit einem deutlichen Anstieg der Hausarbeit und Kinderbetreuungszeit sowie einer Abnahme an Erwerbsstunden verbunden, und zwar unabhängig vom Haushaltseinkommen und der Ressourcenkonstellation vor der Geburt (Dechant et al. 2014; Kühhirt 2012).

Die Persistenz der traditionellen Arbeitsteilung und des Erwerbsarrangements trotz Veränderungen in der Ressourcenausstattung von Frauen wird mithilfe von soziologischen Ansätzen erklärt. So argumentiert der Doing-Gender-Ansatz (West und Zimmerman 1987), dass Männer es vermeiden, Haushaltstätigkeiten zu übernehmen, wenn sie ihre Geschlechteridentität bzw. ihre Position als Familienernährer als gefährdet ansehen (Brines 1994). Dies ist insbesondere dann der Fall, wenn die Frau eine höhere ökonomische Verhandlungsmacht als ihr Partner hat. Aufgrund der beobachteten zunehmenden Unsicherheiten auf dem Arbeitsmarkt seit den 1990er-Jahren, die sich auch in den Erwerbsverläufen von Männern bemerkbar machen (Simonson et al. 2015), lässt sich vermuten, dass Männer ihre Erwerbsidentität als gefährdet ansehen und daher weniger Haushalts- und Familienarbeit übernehmen. Zudem setzt die Politik ambivalente Anreize für Elternpaare, die Haushalts- und Familienarbeit nach „modernen“ (z. B. Einführung der „Vätermonate“) und „traditionellen“ (z. B. Ehegattensplitting) Geschlechterparadigmen auszugestalten (Grunow 2016). Diese Aspekte hängen mit der seit Jahrzehnten beobachteten „stalled revolution“ zusammen (Hochschild und Machung 1989), welche die Langsamkeit des Geschlechterrollenwandels und die Ungleichheit der Geschlechterrollenverhältnisse hervorhebt.

Jedoch geben Studien auch Hinweise auf eine Konvergenz der Erwerbsverläufe zwischen Müttern und Vätern: Während Väter, die vor 1960 geboren wurden, zur Erhöhung von Arbeitsstunden nach der Geburt eines Kindes neigen, tendieren Väter, die nach 1960 geboren wurden, eher zu einer Reduktion von Arbeitsstunden (Choi et al. 2008; Pollmann-Schult und Reynolds 2017). Kaufman und Uhlenberg (2000) beschreiben diese Entwicklung als den Übergang vom Good-provider-Modell zum Involved-father-Modell. Während für Väter laut Good-provider-Modell nach der Geburt eines Kindes ihre Erwerbsidentität im Vordergrund steht und sie als Ausdruck ihrer Vaterrolle für die Sicherung des Familienunterhalts zur Mehrarbeit tendieren, passen Väter laut Involved-father-Modell ihre Erwerbsidentität an die Familienidentität an (ähnlich wie Mütter), und sind im höheren Maße in Familientätigkeiten involviert. Passend dazu finden jüngere Studien einen Zusammenhang zwischen der Elterngeldreform des Jahres 2007 und einer erhöhten Kinderbetreuungszeit durch Väter in den ersten Monaten nach der Kindsgeburt (Schober 2013; Tamm 2019; Geisler und Kreyenfeld 2019). Zudem wurde gezeigt, dass die Aufnahme von Elternzeit durch Väter die Arbeitsmarktbeteiligung und Arbeitsstunden der Partnerinnen kurzfristig erhöhte (Tamm 2019). Die wachsende Selbstverständlichkeit der Erwerbsbeteiligung von Frauen sowie die betonte Relevanz der Gleichstellung von Frauen und Männern in der öffentlichen Debatte scheint sich somit nicht nur im Wandel der *Vorstellungen* egalitärer Arbeitsteilung, sondern auch tatsächlich im *Verhalten *zu manifestieren. Es findet sich ein allgemeiner Trend hin zur Verringerung des Unterschieds zwischen den Zeitinvestitionen in die Erwerbstätigkeit und die Kinderbetreuung zwischen Müttern und Vätern (Bianchi et al. 2006; Dribe und Stanfors 2009). Dieser Trend zeigt sich auch in Deutschland speziell für hochgebildete Personen (Brandt 2019).

Weiterhin sind Ausdifferenzierungen der Erwerbsverläufe im Zusammenhang mit den Tendenzen zur Pluralisierung und zunehmender Diskontinuität zu erwarten. Seit Beginn der 1990er-Jahre haben insbesondere die fortschreitende Globalisierung und die sich verändernde Arbeitsmarktsituation im Zuge der Wiedervereinigung von Ost- und Westdeutschland zu Veränderungen von Erwerbsverläufen geführt (Simonson et al. 2011). Die Arbeitsmarktflexibilisierung der 2000er-Jahre hat zur Steigerung von nichtstandardisierten sowie atypischen Beschäftigungsformen beigetragen (Sperber und Walwei 2015; Haller und Jahn 2014). Erwerbsverläufe werden somit einerseits unterschiedlicher und differenzieren sich aus (Pluralisierung) und sind andererseits durch eine zunehmende Anzahl von Übergängen gekennzeichnet (zunehmende Diskontinuität). Die zunehmenden Diskontinuitäten äußern sich bei Männern häufig in Verkürzungen von Erwerbszeiten und einer Zunahme an Nichterwerbstätigkeitszeiten, speziell an Arbeitslosigkeit. Bei Frauen zeigen sich Diskontinuitäten hingegen in Verkürzungen der Zeiten in Haushaltstätigkeiten und in der Zunahme an Erwerbszeiten, hauptsächlich durch Teilzeittätigkeit (Simonson et al. 2011; Trischler 2012). Somit kann eine Tendenz zur Konvergenz der Paarerwerbsverläufe nach einem Übergang in die Elternschaft auch mit den zunehmenden Diskontinuitäten zusammenhängen, die nicht unbedingt auf den Übergang in die Elternschaft per se zurückzuführen sind.

### Forschungshypothesen

Bezugnehmend auf die oben herausgearbeiteten theoretischen Ansätze sowie aufgrund der familien- und sozialpolitischen Reformen werden insgesamt vier Hypothesen formuliert. Gemäß haushaltsökonomischen und ressourcentheoretischen Ansätzen ist zu erwarten, dass die traditionelle Arbeitsteilung im Sinne eines Alleinverdienermodells aufgrund der gestiegenen Humankapitalausstattung von Frauen und der damit verknüpften verbesserten Erwerbschancen für die hier betrachteten Kohorten an Bedeutung verloren hat.

#### H 1   

Die traditionellen Erwerbsarrangements in den ersten drei Jahren nach dem Übergang in die Elternschaft verlieren im Kohortenvergleich an Bedeutung.

Zudem wird angenommen, dass im Zuge des Wertewandels mit sich verändernden Rollenvorstellungen von Frauen und Männern, wie z. B. dem Übergang vom Good-provider-Modell zum Involved-father-Modell, und politischen Interventionen im Kohortenvergleich eine Konvergenz in den partnerschaftlichen Erwerbsverläufen zwischen Frauen und Männern eintritt. Dies kann einerseits mit der verstärkten Erwerbsbeteiligung von Müttern und Haushalts- sowie Elternzeiten von Vätern im Zusammenhang stehen. Andererseits können die Entwicklungen in den Erwerbsverläufen vermehrt auf die zunehmenden Unsicherheiten auf dem Arbeitsmarkt oder auf zunehmende Bildungszeiten zurückgeführt werden.

#### H 2.1

Die Erwerbskonstellationen konvergieren im Kohortenvergleich, indem Väter immer mehr Haushalts- und Elternzeiten und Mütter immer mehr Erwerbstätigkeitszeiten in den ersten Monaten nach dem Übergang in die Elternschaft aufweisen.

#### H 2.2

Die Erwerbskonstellationen konvergieren im Kohortenvergleich, wobei insbesondere bei Vätern die sonstigen Nichterwerbstätigkeitsphasen zunehmen, was die generelle Tendenz zu zunehmenden Diskontinuitäten widerspiegelt.

Angesichts der sich wandelnden Geschlechteridentitäten und Rollenvorstellungen ist zu vermuten, dass der Arbeitsteilung vor dem Übergang in die Elternschaft eine immer größere Bedeutung für die Erwerbsarrangements danach zukommt. Somit kann erwartet werden, dass für die jüngere Kohorte die moderne oder egalitäre partnerschaftliche Arbeitsteilung vor dem Übergang in die Elternschaft eine größere Rolle für die Wahrscheinlichkeit moderner oder egalitärer Erwerbsarrangements nach diesem Übergang spielt als dies noch bei den älteren Kohorten der Fall war.

#### H 3   

Vorgeburtliche moderne oder egalitäre partnerschaftliche Arbeitsteilung geht im Kohortenvergleich immer deutlicher mit den nachgeburtlichen modernen oder egalitären Erwerbsarrangements einher.

## Empirische Analyse

### Daten und Methoden

Als Datengrundlage der Analysen dienen die Daten des Sozio-oekonomischen Panels (SOEP).^2^ Das SOEP ist eine repräsentative Wiederholungsbefragung privater Haushalte in Deutschland, die seit 1984 in Westdeutschland und seit 1990 in Ostdeutschland jährlich durchgeführt wird (Goebel et al. 2019). Für die vorliegende Analyse verwenden wir Daten aus der Welle 36 vom Jahr 1984 bis zum Jahr 2019. Aufgrund der Datenlage werden ausschließlich zwei Geschlechter berücksichtigt sowie ausschließlich Partnerschaften zwischen Frauen und Männern. Da Prozesse der partnerschaftlichen Arbeitssteilung im Haushalt nicht auf Einheiten (Monaden), sondern Zweiheiten (Dyaden) basieren, werden in unserer Analyse sowohl die Information der Frauen als auch die Information der Partner berücksichtigt (Dyadische Daten). Das ist mit dem SOEP möglich, indem die Informationen der Partnerinnen mit denen der Partner über eine Identifikationsnummer zusammengeführt werden können. Wir betrachten drei Geburtskohorten (Kohorte 1970–1974, Kohorte 1975–1979 und Kohorte 1980–1984), wobei es sich um die Geburtsjahre der Frauen handelt. Unser Analysesample setzt sich aus den Fällen zusammen, für die (1) Befragungsdaten zu einer Partnerschaft vorliegen; für die (2) der Zeitpunkt der Geburt des ersten Kindes monatsgenau feststellbar ist und für die (3) Informationen zu Erwerbsverläufen von Frauen und ihren Partnern über drei Jahre hinweg – von der Geburt bis 36 Monate danach – bezogen werden können. Nach dieser Spezifikation verbleiben 900 Paare in der Analyse; 242 davon gehören Kohorte 1970–1974, 354 Kohorte 1975–1979 und 304 Kohorte 1980–1984 an. Das Durchschnittsalter bei der Geburt des ersten Kindes liegt für Frauen bei 27,4 Jahren und bei ihren Partnern bei 29,5 Jahren.

Für die Beschreibung der Erwerbskonstellationen im partnerschaftlichen Kontext wird die Methode der Sequenzmusteranalyse verwendet. Während herkömmliche längsschnittliche regressionsanalytische Verfahren wie Ereignisanalysen bestimmte Ereignisse (z. B. Geburt eines Kindes) oder Übergänge (z. B. von Erwerbs- in die Nichterwerbstätigkeit) in den Fokus stellen, haben Sequenzmusteranalysen den entscheidenden Vorteil, dass sie den Verlauf (in unserem Fall den Erwerbsverlauf) in seiner gesamten Komplexität in den Vordergrund stellen. Somit ist die Methode der Sequenzmusteranalyse in der Lage, die Anzahl, die Reihenfolge und auch die Dauer der interessierenden Status in der Analyse zu erfassen und zu berücksichtigen (Aisenbrey und Fasang 2010, Zimmermann und Konietzka 2020). Allerdings ist anzumerken, dass es sich hierbei um ein beschreibendes und kein erklärendes Verfahren handelt. Nichtsdestotrotz ermöglicht die Sequenzmusteranalyse eine detaillierte Untersuchung der Erwerbskonstellationen im partnerschaftlichen Kontext. Da in der Analyse kombinierte partnerschaftliche Erwerbsverläufe im Zentrum stehen, reduzieren wir die Anzahl an möglichen Kombinationen, indem die Selbstangaben zur Erwerbstätigkeit (Vollzeit- und Teilzeittätigkeit), Elternzeit/Haushaltstätigkeit und sonstige Nichterwerbstätigkeit (Arbeitslosigkeit, Bildung bzw. Weiterbildung und Sonstiges) zusammengefasst betrachtet werden. Somit liegt das Augenmerk der Analyse darauf, wie sich Erwerbstätigkeit und Elternzeit/Haushaltstätigkeit im Wandel auf die Elternteile verteilt. Insgesamt ergeben sich neun Erwerbskonstellationen:

1. Frau erwerbstätig, Partner erwerbstätig;
2. Frau erwerbstätig, Partner Haushaltstätigkeit/Elternzeit;
3. Frau erwerbstätig, Partner sonstig nicht erwerbstätig;
4. Frau Haushaltstätigkeit/Elternzeit, Partner erwerbstätig;
5. Frau Haushaltstätigkeit/Elternzeit, Partner Haushaltstätigkeit/Elternzeit;
6. Frau Haushaltstätigkeit/Elternzeit, Partner sonstig nicht erwerbstätig;
7. Frau sonstig nicht erwerbstätig, Partner erwerbstätig;
8. Frau sonstig nicht erwerbstätig, Partner Haushaltstätigkeit/Elternzeit;
9. Frau sonstig nicht erwerbstätig, Partner sonstig nicht erwerbstätig.

Mithilfe einer Clusteranalyse wird eine Typisierung partnerschaftlicher Erwerbsverläufe vorgenommen. Hierfür werden die einzelnen Sequenzen anhand des Lesnard’s dynamic Hamming miteinander verglichen: Der Algorithmus berechnet paarweise Distanzen für alle Sequenzen an jedem gegebenen Zeitpunkt und vergleicht somit alle partnerschaftlichen Verläufe einen Monat nach der Geburt, zwei Monate nach der Geburt und so weiter. Somit ist Lesnard’s dynamic Hamming im Vergleich zu herkömmlichen Algorithmen besser in der Lage, die Zeitabhängigkeit der beobachteten Zustände über den Verlauf zu berücksichtigen (für weitere Informationen siehe Aisenbrey und Fasang 2010, Lesnard 2010). Anschließend werden die gebildeten Distanzen zu einer Gesamtdistanz zusammengefasst, welche für die Bildung von Clustern verwendet wird. Die Clusterbildung erfolgt empirisch. Wir verwenden eine hierarchische Clusteranalyse und machen Gebrauch von der Ward’s linkage-Methode, die den Vorteil hat, tendenziell ähnlich große Cluster zu generieren (Ward 1963). Grundsätzlich erfolgt die Clusterbildung nach dem Prinzip, die Distanzen oder Unähnlichkeiten zwischen den Erwerbsverläufen in den Clustern zu minimieren und zwischen den Clustern zu maximieren. Anhand des Elbow-Kriteriums können wir anschließend die Clusteranzahl festlegen. Die bestgeeignete Clusteranzahl scheint bei vier zu liegen (siehe Abb. 1 im Online-Anhang): Die Fehlerquadratsumme (oder die Unähnlichkeit der Verläufe in den Clustern) verringert sich am deutlichsten von eins auf zwei, zwei auf drei und drei auf vier Cluster. Nach vier Clustern verringert sich die Fehlerquadratsumme weiterhin, aber nicht in dieser Deutlichkeit.

Im Anschluss der Sequenz- und Clusteranalyse wird eine multinomiale logistische Regression durchgeführt. Die vier Cluster dienen in der Analyse als abhängige Variablen. Um die Frage zu beantworten, inwiefern sich ein Wandel von Erwerbskonstellationen im Partnerschaftskontext beobachten lässt, werden die Geburtskohorten als unabhängige Variablen verwendet (Kohorte 1970–1974 dient als Referenz). Als unabhängige Variablen werden zudem Informationen zur partnerschaftlichen Arbeitsteilung vor der Geburt des ersten Kindes einbezogen, um deren Rolle für die nachgeburtlichen Erwerbsarrangements im Wandel zu beleuchten. Dabei werden zwei Dummysets gebildet, die jeweils angeben, wie die Aufteilung im Haushalt sowie in der Erwerbsarbeit vor der Geburt war (Frau < Partner (Referenz für die Arbeitsteilung im Erwerb); Frau ≈ Partner; Frau > Partner (Referenz für die Arbeitsteilung im Haushalt). In der Kategorie „Frau < Partner“ übernimmt die Frau weniger als 35 % der Tätigkeit; in der Kategorie „Frau ≈ Partner“ übernehmen beide zwischen 35 und 65 % der Tätigkeit; in der Kategorie „Frau > Partner“ übernimmt Frau mehr als 65 % der Tätigkeit. Weiterhin fließt eine Reihe an Kontrollvariablen in die Analyse mit ein. In der Analyse wird das Alter (metrisch) und der Bildungsstand (Dummyset, niedrig (Referenz), mittel und hoch) von Frauen und ihren Partnern bei Geburt kontrolliert. Hiermit soll dem Umstand unterschiedlicher Altersstrukturen je nach Geburtskohorte Rechnung getragen werden. Darüber hinaus berücksichtigen wir die Region (Ostdeutschland = 1; Westdeutschland = 0), da historisch begründet Elternpaare aus Westdeutschland zu traditionelleren Erwerbskonstellationen neigen könnten als Elternpaare aus Ostdeutschland (Kreyenfeld und Geisler 2006). Ferner wird der Familienstand (verheiratet = 1; nicht verheiratet = 0) berücksichtigt, da der Übergang in eine Ehe ein weiteres kritisches Ereignis für die Ausübung traditioneller Arbeitsteilung sein kann (Frommert und Strauß 2013). Außerdem kontrollieren wir, ob in der Beobachtungszeit von 36 Monaten mindestens ein weiteres Kind geboren wurde, da dies Auswirkungen auf die Zugehörigkeit zu einem bestimmten Cluster haben könnte. Mehr Informationen zu den unabhängigen Variablen finden sich in Tab. 1 im Online-Anhang. Für die Auswertung der Ergebnisse der Regressionsanalyse weisen wir Average Marginal Effects (AME) aus. Die AME geben die durchschnittliche Veränderung der Wahrscheinlichkeit (gemittelt über alle Beobachtungen) des interessierenden Ereignisses in Prozentpunkten wieder, wenn sich der betreffende Prädiktor marginal bzw. um eine Einheit erhöht.

### Ergebnisse

#### Sequenzmuster- und Clusteranalyse

Tabelle 1 zeigt zunächst, wie die Erwerbskonstellationen in den ersten 36 Monaten nach dem Übergang in die Elternschaft über die drei betrachteten Kohorten hinweg verteilt sind. Für alle Kohorten ist zu beobachten, dass die traditionelle Konstellation Haushaltstätigkeit/Elternzeit (Frau) – Erwerbstätigkeit (Partner) dominiert, wobei sie wie erwartet über die Kohorten hinweg eine Bedeutungsabnahme erfährt. Während die Paare der Kohorte 1970–1974 noch etwas über 23 Monate in diesem Status verbracht haben, waren es für die Paare der Kohorte 1980–1984 knapp 17 Monate. Hingegen gewinnt die Konstellation der Erwerbstätigkeit beider Elternteile an Bedeutung, und zwar von knapp 7 Monate bei Kohorte 1970–1974 auf fast 9 Monate bei Kohorte 1980–1984. Auch viele Konstellationen mit sonstiger Nichterwerbstätigkeit nehmen an Bedeutung zu. Dies kann ein Hinweis auf die steigende Diskontinuität der Erwerbsverläufe sein. Nicht zu beobachten ist hingegen eine Bedeutungszunahme an Konstellationen, in denen die Partner die Haushaltstätigkeit/Elternzeit übernehmen. Eine Ausnahme stellt die Konstellation beider Elternteile in Haushaltstätigkeit/Elternzeit dar, die über die Kohorten hinweg leicht zunimmt (die durchschnittliche Dauer liegt für alle Kohorten unter einem Monat). Darüber hinaus weist Kohorte 1980–1984 im Vergleich zu den beiden älteren Kohorten eine höhere durchschnittliche Anzahl an Elementen (monatliche Status) und Episoden (Phasen in einem bestimmten monatlichen Status) auf, was wiederum für eine Destandardisierung der partnerschaftlichen Erwerbsverläufe spricht (siehe Tab. 2 im Online-Anhang).

**Table 1:** 

	Kohorte 1970–1974	Kohorte 1975–1979	Kohorte 1980–1984
*Partnerschaftliche Erwerbskonstellation, Dauer in Monaten (Frau – Partner)*			
Haushaltstätigkeit/Elternzeit – Erwerbstätigkeit	23,03	22,21	16,88
Haushaltstätigkeit/Elternzeit – Haushaltstätigkeit/Elternzeit	0,32	0,89	0,66
Haushaltstätigkeit/Elternzeit – Nichterwerbstätigkeit	2,55	1,95	4,55
Erwerbstätigkeit – Erwerbstätigkeit	6,68	8,34	8,80
Erwerbstätigkeit – Haushaltstätigkeit/Elternzeit	0,81	0,26	0,23
Erwerbstätigkeit – Nichterwerbstätigkeit	0,82	0,39	0,88
Nichterwerbstätigkeit – Erwerbstätigkeit	1,37	1,40	3,35
Nichterwerbstätigkeit – Haushaltstätigkeit/Elternzeit	0,02	0,07	0,12
Nichterwerbstätigkeit – Nichterwerbstätigkeit	0,40	0,50	1,08
Gesamtlänge	36,00	36,00	36,00
*Fallzahl*	*242*	*354*	*304*

Quelle: SOEP v36, gewichtete Ergebnisse, eigene Darstellung

In Tab. 2 sind die Erwerbskonstellationen nach Clustern angegeben. Im Online-Anhang (Abb. 2) finden sich außerdem die Indexplots für die einzelnen Cluster, die nicht nur die Erwerbskonstellationen in den jeweiligen Clustern angeben, sondern auch die Abfolge der Übergänge in den Clustern nachvollziehen. *Cluster 1 „Traditionelle Erwerbsarrangements“* ist eindeutig von der Haushaltstätigkeit und Elternzeit der Frauen und Erwerbstätigkeit von Männern dominiert. *Cluster 2 „Abgeschwächte traditionelle Erwerbsarrangements“* ist durch die Haushaltstätigkeit und Elternzeit bzw. Nichterwerbstätigkeit von Frauen und die Erwerbstätigkeit von Partnern dominiert. Einige Verläufe sind außerdem durch spätere Einstiege von Frauen in die Erwerbstätigkeit gekennzeichnet. Bei einigen anderen Verläufen steigen Frauen zwischendurch in die Erwerbstätigkeit ein und verlassen sie dann wieder zugunsten einer Haushaltstätigkeit bzw. Elternzeit. Ausschlaggebend hierfür könnte die Geburt eines weiteren Kindes sein. *Cluster 3 „Doppelte Erwerbstätigkeit“* ist durch die Erwerbstätigkeit beider Elternteile dominiert. Dabei ist aus der existierenden Forschung bekannt, dass es sich bei Müttern häufig um eine Teilzeitbeschäftigung handelt, während Väter meist in Vollzeit erwerbstätig sind (Gallego Granados et al. 2019). In diesem Cluster ereignen sich viele Übergänge von Frauen in die Erwerbstätigkeit nach einer Phase der Haushaltstätigkeit/Elternzeit ein bis zwei Jahre nach der Geburt des ersten Kindes. *Cluster 4 „Diskontinuierliche Erwerbstätigkeit“* ist durch unbeständige Erwerbsverläufe bestimmt. Hier kommen die Konstellationen Haushaltstätigkeit/Elternzeit von beiden Elternteilen im Vergleich aller Cluster am häufigsten vor. Dennoch sind auch hier andere Konstellationen bedeutend (z. B. Haushaltstätigkeit/Elternzeit (Frau) – Nichterwerbstätigkeit (Partner)), die eher sonstige Nichterwerbstätigkeit von Partnern beinhalten und nicht primär mit Haushaltstätigkeit oder Elternzeit zu tun haben müssen. Die Nummer an Elementen und Episoden verdeutlicht zusätzlich (siehe Tab. 2 im Online-Anhang), dass Cluster 4 die höchste Anzahl an Elementen und Episoden aufweist und somit als Cluster mit diskontinuierlichen partnerschaftlichen Erwerbsverläufen interpretiert werden kann. Das Cluster mit den stabilsten partnerschaftlichen Erwerbsverläufen ist mit Abstand Cluster 1.

**Table 2:** 

	Cluster 1	Cluster 2	Cluster 3	Cluster 4
*Partnerschaftliche Erwerbskonstellation, Dauer in Monaten (Frau – Partner)*				
Haushalt/Elternzeit – Erwerbstätigkeit	32,98	20,03	11,90	4,23
Haushalt/Elternzeit – Haushalt/Elternzeit	0,42	0,62	0,56	1,53
Haushalt/Elternzeit – Nichterwerbstätigkeit	0,74	1,52	0,19	17,19
Erwerbstätigkeit – Erwerbstätigkeit	0,78	7,06	21,87	3,66
Erwerbstätigkeit – Haushalt/Elternzeit	0,03	0,36	0,56	1,20
Erwerbstätigkeit – Nichterwerbstätigkeit	0,04	0,63	0,24	3,21
Nichterwerbstätigkeit – Erwerbstätigkeit	0,94	4,86	0,67	1,54
Nichterwerbstätigkeit – Haushalt/Elternzeit	0,04	0,05	0,00	0,34
Nichterwerbstätigkeit – Nichterwerbstätigkeit	0,04	0,87	0,00	3,11
Gesamtlänge	36,00	36,00	36,00	36,00
*Fallzahl*	*319*	*238*	*229*	*114*

Quelle: SOEP v36, gewichtete Ergebnisse, eigene Darstellung

#### Multivariate Analysen

Tabelle 3 zeigt die Ergebnisse einer multinomialen logistischen Regression (siehe Tab. 3 im Online-Anhang für die Ergebnisse mit Kontrollvariablen). Modelle M1.1, M2.1, M3.1 und M4.1 zeigen die Wahrscheinlichkeit der Clusterzugehörigkeit nach Kohorten unter Kontrolle von Alter (Frau und Partner), Bildung (Frau und Partner), Familienstatus, Geburt weiterer Kinder im Beobachtungszeitraum und Region. In den Modellen M1.2, M2.2, M3.2 und M4.2 werden zusätzlich Variablen für die Arbeitsteilung im Haushalt und in der Erwerbstätigkeit vor der Geburt des ersten Kindes einbezogen. Das Ziel hierbei ist zu untersuchen, ob die Hinzunahme dieser Information Auswirkung auf die Wahrscheinlichkeiten der Zugehörigkeit zu einem Cluster hat.

**Table 3:** 

	Cluster 1		Cluster 2		Cluster 3		Cluster 4	
	M1.1	M1.2	M2.1	M2.2	M3.1	M3.2	M4.1	M4.2
	AME *(se)*	AME *(se)*	AME *(se)*	AME *(se)*	AME *(se)*	AME *(se)*	AME *(se)*	AME *(se)*
Kohorte 1975–1979	−0,07+ *(0,04)*	−0,08* *(0,04)*	0,04 *(0,04)*	0,03 *(0,04)*	0,03 *(0,04)*	0,04 *(0,04)*	−0,00 *(0,03)*	0,01 *(0,03)*
Kohorte 1980–1984	−0,20** *(0,04)*	−0,20** *(0,04)*	0,05 *(0,04)*	0,04 *(0,04)*	0,10** *(0,04)*	0,11** *(0,04)*	0,05+ *(0,03)*	0,05 *(0,03)*
Arbeitsteilung Haushalt: Frau ≈ Partner	–	−0,05 *(0,09)*	–	−0,15* *(0,07)*	–	0,05 *(0,08)*	–	0,15 *(0,10)*
Arbeitsteilung Haushalt: Frau < Partner	–	−0,13*** *(0,04)*	–	−0,02 *(0,04)*	–	0,00 *(0,03)*	–	0,15*** *(0,04)*
Arbeitsteilung Erwerb: Frau ≈ Partner	–	−0,02 *(0,04)*	–	−0,08* *(0,04)*	–	0,10** *(0,03)*	–	0,01 *(0,03)*
Arbeitsteilung Erwerb: Frau > Partner	–	0,01 *(0,06)*	–	−0,18*** *(0,04)*	–	−0,09+ *(0,05)*	–	0,25*** *(0,07)*
Pseudo R2	0,105	0,144	0,105	0,144	0,105	0,144	0,105	0,144
*Fallzahl*	*900*	*900*	*900*	*900*	*900*	*900*	*900*	*900*

Kontrollvariablen: Region (Ost‑/Westdeutschland); Familienstatus, Geburt weiteren Kindes, Alter (Frau und Partner), Bildung (Frau und Partner). Es wurden Kategorien mit fehlenden Werten gebildet und in die Analyse miteinbezogen (in der Tabelle nicht dargestellt); somit bleibt die Fallzahl bei 900 Fällen. Quelle: SOEP v36, eigene Darstellung

*AME* Durchschnittliche marginale Effekte, *(se)* Standardfehler

*** *p* < 0,001 **p < 0,01 *p < 0,05 +*p* < 0,10

Für Cluster 1 „Traditionelle Erwerbsarrangements“, der durch Haushaltstätigkeit/Elternzeit von Frauen und Erwerbstätigkeit von Partnern gekennzeichnet ist, beobachten wir für Kohorte 1975–1979 und insbesondere für Kohorte 1980–1984 (im Vergleich zu Kohorte 1970–1974) eine deutlich verringerte Zugehörigkeitswahrscheinlichkeit (M1.1). Dieses Ergebnis verändert sich durch die Berücksichtigung der Arbeitsteilung vor der Geburt des ersten Kindes nur unwesentlich (M1.2). Das heißt, dass die Arbeitsteilung vor der Erstgeburt zwar eine Rolle spielt (es gibt eine um 13 Prozentpunkte geringere Zugehörigkeitswahrscheinlichkeit zu Cluster 1, wenn die Frau vor dem Übergang in die Elternschaft weniger Haushaltsaufgaben übernommen hat als der Partner), der Kohorteneffekt bleibt davon aber unberührt. Für Cluster 2 „Abgeschwächte traditionelle Erwerbsarrangements“, der durch die Erwerbstätigkeit der Partner und die Haushaltstätigkeit/Elternzeit bzw. sonstige Nichterwerbstätigkeit der Frauen gekennzeichnet ist, können keine statistisch signifikanten Unterschiede bezüglich der Zugehörigkeitswahrscheinlichkeit nach Kohorten (M2.1) festgestellt werden. Bei egalitärer oder moderner Arbeitsteilung vor der Geburt eines Kindes ist die Wahrscheinlichkeit geringer, diesem Cluster anzugehören (M2.2). Für Cluster 3 „Doppelte Erwerbstätigkeit“, der durch die Erwerbstätigkeit beider Elternteile gekennzeichnet ist, zeigt sich für die jüngste Kohorte eine höhere Zugehörigkeitswahrscheinlichkeit im Vergleich zu der ältesten Kohorte (M3.1). Dieses Ergebnis bleibt auch nach Kontrolle der Arbeitsteilung vor der Erstgeburt bestehen (M3.2). Damit bleibt auch hier der Kohorteneffekt trotz Berücksichtigung der Arbeitsteilung vor der Geburt des ersten Kindes stabil. Dem Cluster 4 „Diskontinuierliche Erwerbstätigkeit“ gehört die Kohorte 1980–1984 mit einer etwas höheren Wahrscheinlichkeit an als die Kohorte 1970–1974 (M4.1). Zwischen den Kohorten 1970–1974 und 1975–1979 gibt es hingegen keinen statistisch signifikanten Unterschied. Wird die Arbeitsteilung vor der Geburt des ersten Kindes berücksichtigt, ist der in M4.1 beobachtete Unterschied zwischen der jüngsten und der ältesten Kohorte nicht mehr statistisch signifikant (M4.2). Für die Arbeitsteilung vor der Erstgeburt zeigt sich, dass bei moderner Arbeitsteilung im Haushalt und in der Erwerbstätigkeit die Wahrscheinlichkeit steigt, dem diskontinuierlichen Cluster 4 anzugehören. Die Arbeitsteilung vor der Kindsgeburt scheint somit eine höhere Erklärungskraft für die Zugehörigkeit zu diesem Cluster zu haben als die Zugehörigkeit zu den einzelnen Kohorten. Allerdings muss betont werden, dass in M4.1 der AME für Kohorte 1980–1984 auf einem zehnprozentigen Signifikanzniveau bereits nur schwach statistisch signifikant war.

Bezugnehmend auf die Hypothesen kann anhand dieser Ergebnisse Hypothese *H 1* („Die traditionellen Erwerbsarrangements in den ersten drei Jahren nach dem Übergang in die Elternschaft verlieren im Kohortenvergleich an Bedeutung“) bestätigt werden: Bei der Zugehörigkeit zum Cluster 1 „Traditionelle Erwerbsarrangements“ ist über die Kohorten hinweg eine deutliche Abnahme zu beobachten. Bei der Gesamtbetrachtung unabhängig von den einzelnen Clustern (siehe Tab. 1) zeigt sich jedoch, dass die Erwerbsverläufe 36 Monate nach dem Übergang in die Elternschaft auch für die jüngste Kohorte ganz klar durch die Erwerbstätigkeit der Partner und Haushaltstätigkeit/Elternzeit der Frauen dominiert sind. Auch bei Cluster 2 „Abgeschwächte traditionelle Erwerbsarrangements“ ist diese Konstellation dominant (siehe Tab. 2). Cluster 1 und Cluster 2 machen zusammen über 60 % aller Verläufe aus. Daher verliert die traditionelle Arbeitsteilung nach der Geburt des ersten Kindes über die beobachteten Kohorten hinweg zwar weiterhin an Bedeutung, bleibt dennoch die häufigste Konstellation.

Hypothese *H 2.1* („Die Erwerbskonstellationen konvergieren im Kohortenvergleich, indem Väter immer mehr Haushalts- und Elternzeiten und Mütter immer mehr Erwerbstätigkeitszeiten in den ersten Monaten nach dem Übergang in die Elternschaft aufweisen“) kann abgelehnt und Hypothese *H 2.2* („Die Erwerbskonstellationen konvergieren im Kohortenvergleich, wobei insbesondere bei Vätern die sonstigen Nichterwerbstätigkeitsphasen zunehmen, was die generelle Tendenz zu zunehmenden Diskontinuitäten widerspiegelt“) bestätigt werden: Die Konvergenz der partnerschaftlichen Erwerbsverläufe nach dem Übergang in die Elternschaft findet einerseits Ausdruck darin, dass die jüngste Kohorte mit einer höheren Wahrscheinlichkeit Cluster 3 „Doppelte Erwerbstätigkeit“ zugehörig ist als die älteste Kohorte. Andererseits wird deutlich, dass keine Konvergenz bezüglich der Zeiten in Haushaltstätigkeit/Elternzeit zwischen Frauen und ihren Partnern zu beobachten ist. Zwar sind in Cluster 4 „Diskontinuierliche Erwerbstätigkeit“ die meisten Verläufe mit der Konstellation Haushaltstätigkeit/Elternzeit der Partner zu finden. Dennoch ergeben sich die meisten Diskontinuitäten tatsächlich durch sonstige Nichterwerbstätigkeiten, die vermutlich nicht primär mit der Geburt des ersten Kindes zusammenhängen.

Hypothese *H 3* („Vorgeburtliche moderne oder egalitäre partnerschaftliche Arbeitsteilung geht im Kohortenvergleich immer deutlicher mit den nachgeburtlichen modernen oder egalitären Erwerbsarrangements einher“) kann nicht bestätigt werden: Zwar kann für Cluster 4 „Diskontinuierliche Erwerbstätigkeit“ beobachtet werden, dass eine moderne partnerschaftliche Arbeitsteilung vor der Geburt die Zugehörigkeit zu dem Cluster mit den meisten modernen Erwerbskonstellationen nach der Geburt begünstigt. Dennoch sind, wie oben bereits aufgeführt, viele Verläufe in diesem Cluster nicht auf die erhöhten Haushalts- oder Kinderbetreuungszeiten der Partner zurückzuführen, sondern eher als ein Ausdruck erhöhter Erwerbsdiskontinuitäten zu sehen.

## Diskussion und Schlussbetrachtung

Die Studie untersuchte, inwiefern zwischen Frauen und Männern im Partnerschaftskontext über die Kohorten hinweg eine Konvergenz der Erwerbsverläufe nach dem Übergang in die Elternschaft zu beobachten ist. Da familiäre Entscheidungen über die Erwerbsarrangements in den ersten Jahren nach der Familiengründung eine Grundlage für langfristige geschlechterspezifische Ungleichheiten hinsichtlich weiterer Erwerbs- und Einkommenschancen sowie der Alterssicherung bilden können, ist es wichtig, partnerschaftliche Erwerbskonstellationen in der Phase des Übergangs in die Elternschaft und Veränderungen der Folgen dieses Übergangs über die Zeit hinweg zu untersuchen.

Für die in den 1970er- und 1980er-Jahren Geborenen konnte die Studie zeigen, dass das traditionelle Modell von Erwerbskonstellationen, bei dem Frauen nach der Geburt eines Kindes für einen längeren Zeitraum aufhören zu arbeiten, in den ersten drei Jahren nach dem Übergang in die Elternschaft zunehmend an Bedeutung verliert. Weiterhin war zu beobachten, dass es immer mehr Elternpaare gibt, in denen beide Elternteile relativ schnell nach der Geburt des ersten Kindes wieder in die Erwerbstätigkeit zurückkehren. Diese Entwicklungen können unter anderem mit Reformen wie der Einführung des Elterngeldes oder der verbesserten Kinderbetreuung zusammenhängen. Kaum zu beobachten war allerdings, dass Väter ihre Erwerbstätigkeit zunehmend zugunsten einer Elternzeit oder einer stärkeren Einbindung in Haushaltstätigkeiten einschränken. Somit scheinen die beobachteten Konvergenzen in den Erwerbsverläufen zwischen Müttern und Vätern eher auf die zunehmenden Erwerbsdiskontinuitäten und weniger auf die familien- und sozialpolitischen Interventionen der letzten Jahrzehnte (siehe Abb. 1) zurückzuführen zu sein, was vor dem Hintergrund der umfangreichen familienpolitischen Reformen als ein gesellschaftsrelevantes Ergebnis einzuschätzen ist.

Des Weiteren wurde beleuchtet, welche Rolle die partnerschaftliche Arbeitsteilung vor der Erstgeburt auf die unmittelbare Ausgestaltung der Erwerbskonstellationen danach spielt. Während die Arbeitsteilung vor dem Übergang in die Elternschaft durchaus eine Rolle dafür spielt, wie die Eltern ihre Erwerbsverläufe im Partnerschaftskontext danach gestalten, scheint sie sich über die Kohorten hinweg nicht wesentlich zu verändern. Das heißt, dass auch für jüngere Kohorten, die in den 1980er-Jahren geboren wurden, ähnlich wie für die älteren Kohorten mit dem Übergang in die Elternschaft eine Retraditionalisierung der Erwerbsverläufe festzustellen ist.

Die eher schwach ausgeprägte Konvergenz der Erwerbsverläufe von Elternpaaren über verschiedene Kohorten hinweg könnte also weniger mit der gelebten Arbeitsteilung oder den Einstellungen vor der Geburt des ersten Kindes zu tun haben, sondern vielmehr mit sozialpolitischen Faktoren zusammenhängen (Levy 2018). Die Reformen in den letzten 15 Jahren haben bereits einige Anreize für eine egalitäre partnerschaftliche Arbeitsteilung nach der Geburt eines Kindes gesetzt (Brandt 2017; Samtleben et al. 2019). Um das Ziel zu erreichen, dass sich Väter in gleicher Weise wie Mütter an der Kinderbetreuung beteiligen und die Erwerbstätigkeit gleichgewichtig zwischen den Eltern aufgeteilt wird, sind allerdings noch mehr Anreize für die Inanspruchnahme der Elternzeit durch Väter notwendig. Solche Anreize für eine stärkere Inanspruchnahme der Elternzeit und eine verstärkte Übernahme von Kinderbetreuungsaufgaben durch Väter können allerdings nur dann greifen, wenn sie mit dem weiteren Abbau von geschlechterspezifischen Ungleichheiten am Arbeitsmarkt einhergehen. Solange Frauen für die gleichen Tätigkeiten im Mittel schlechter entlohnt werden als Männer und ihre Aufstiegschancen aufgrund von Diskriminierungsprozessen und höheren Teilzeitquoten geringer sind, bleibt es im Paarkontext in humankapitaltheoretischer Perspektive rational, wenn (überwiegend) die Frau ihre Erwerbstätigkeit unterbricht bzw. reduziert, wodurch ihre haushaltsinterne Verhandlungsposition geschwächt bleibt. Eine egalitäre Arbeitsteilung zwischen Vätern und Müttern kann somit nur im Zuge einer weitergehenden Verringerung geschlechterspezifischer Ungleichheiten auf dem Arbeitsmarkt und im Zusammenspiel mit einem Abbau geschlechterstereotyper Rollenmuster erreicht werden. Hier ist auch die Familien- und Sozialpolitik gefragt, die in Deutschland bislang widersprüchlichen Leitbildern folgt. Gefördert werden einerseits die Erwerbstätigkeit von Frauen im Sinne des Adult-Worker-Modells und die Beteiligung von Vätern an familiären Aufgaben, andererseits entsprechen abgeleitete soziale Sicherungsansprüche in der Kranken- und Rentenversicherung sowie das Ehegattensplitting im Steuerrecht eher dem modernisierten Ernährermodell und stehen einer egalitären Arbeitsteilung entgegen (Peukert 2021).

Um die Analyse handhabbar zu gestalten, haben wir für die kombinierten Erwerbsverläufe die Anzahl an möglichen Kombinationen durch das Zusammenfassen von Status reduziert. Differenzierungen zwischen Vollzeit- und Teilzeittätigkeiten waren dadurch nicht möglich. Dies könnte zur Folge haben, dass in unserer Analyse Veränderungen unterhalb der Differenzierungslinie erwerbstätig – nichterwerbstätig unentdeckt blieben und daher der Wandel in den partnerschaftlichen Erwerbsarrangements zwischen den Kohorten unterschätzt wurde. Unter Einbezug der existierenden Forschung lässt sich dennoch festhalten, dass die Erwerbsquote von Frauen vor allem auf Basis der zunehmenden Teilzeitbeschäftigung zunimmt. Sowohl in West- als auch in Ostdeutschland werden Vollzeitbeschäftigung, aber auch Nichterwerbstätigkeit, von Frauen zunehmend durch Teilzeitarbeit ersetzt (Dieckhoff et al. 2016; Kelle et al. 2017). Für Männer hingegen ist die Zunahme an Teilzeitbeschäftigung nur auf einem sehr niedrigen Niveau zu beobachten (Gallego Granados et al. 2019). Weiterhin kann die vorgelegte Analyse aufgrund der Fokussierung auf die partnerschaftlichen Erwerbsverläufe nach der Geburt des ersten Kindes keinen Aufschluss über das zeitliche Ausmaß und die Veränderung der Beteiligung von Vätern und Müttern an Kinderbetreuung und Haushaltstätigkeiten geben, die auch bei gleichem Erwerbsstatus sehr unterschiedlich ausfallen können. Studien zeigen, dass Väter, die Elternzeit genommen haben, ihre Zeiten für Hausarbeit und Kindererziehung in der Folge erhöhen (Bünning 2016; Schober und Zoch 2019; Tamm 2019). Klünder und Meier-Gräwe (2018) zeigen allerdings auf der Basis von Zeitverwendungsdaten, dass Care-Arbeit von Eltern unabhängig vom Erwerbsumfang der Frau nach wie vor geschlechtsdifferenzierend aufgeteilt und stärker von Müttern als von Vätern geleistet wird. In die gleiche Richtung weisen die Befunde von Hobler et al. (2017), die zeigen, dass selbst zwischen vollzeitbeschäftigten Frauen und Männern sich die geschlechterspezifische Lücke bei Haus- und Fürsorgearbeit finden lässt, was auf nach wie vor persistierende Rollenvorstellungen verweist und deutlich macht, dass bei der innerfamiliären Arbeitsteilung auch außerökonomische Aspekte von Bedeutung sind.

Unsere Befunde zeigen, wie sich partnerschaftliche Erwerbsarrangements in den ersten drei Jahren nach dem Übergang in die Elternschaft in den drei Geburtskohorten 1970–1974, 1975–1979 und 1980–1984 entwickelt haben. Die Frage, wie sich die partnerschaftlichen Erwerbsmuster bei jüngeren Kohorten als den hier untersuchten weiterentwickeln werden, kann mit den vorliegenden Daten noch nicht untersucht werden und bleibt weiterer Forschung vorbehalten. Auch hinsichtlich der Frage, wie sich die Erwerbskonstellationen in den drei Jahren nach der Geburt des Kindes auf die darauffolgenden Erwerbsverläufe sowie auf die Alterssicherung auswirken, sind weitere Forschungsarbeiten notwendig. Die hier vorgelegten Ergebnisse lassen allerdings auch in dieser Hinsicht eher graduelle Veränderungen in den weiteren Erwerbs- und Einkommenschancen als eine deutliche Abkehr von alten Mustern erwarten.

## Supplementary Information





## Footnotes

1 Dargestellt sind nur ausgewählte, nicht alle im Text beschriebene Reformen.

2 In dieser Studie werten wir öffentlich zugängliche Datensätze aus. Die Daten unterliegen Bestimmungen, die ihre Verwendung auf wissenschaftliche Zwecke begrenzen. Die SOEP-Daten sind auf Anfrage nach Abschluss eines kostenlosen Vertrags mit dem DIW erhältlich. Der in der aktuellen Studie verwendete Code ist für alle interessierten Forscherinnen und Forscher auf Anfrage bei der korrespondierenden Autorin erhältlich.
