# Supplementary material for: What Happens After Childbirth? Changes in Partners’ Employment Patterns Three Years After the Transition to Parenthood
Source: Kolner Z Soz Sozpsychol. 2022 Oct 21;74(3):329–51. [Article in German] doi: 10.1007/s11577-022-00860-5 (PMC9589713; doi:10.1007/s11577-022-00860-5)
Supplement: Supplementary file 1 [file 11577_2022_860_MOESM1_ESM.docx]

**Kind – und dann? Wandel partnerschaftlicher Erwerbsverläufe drei Jahre nach dem Übergang in die Elternschaft**

**Nadiya Kelle · Laura Romeu Gordo · Julia Simonson**

**Online-Anhang**

**Online-Anhang**

**Abbildung 1:** Elbow-Kriterium zur Bestimmung der Clusteranzahl


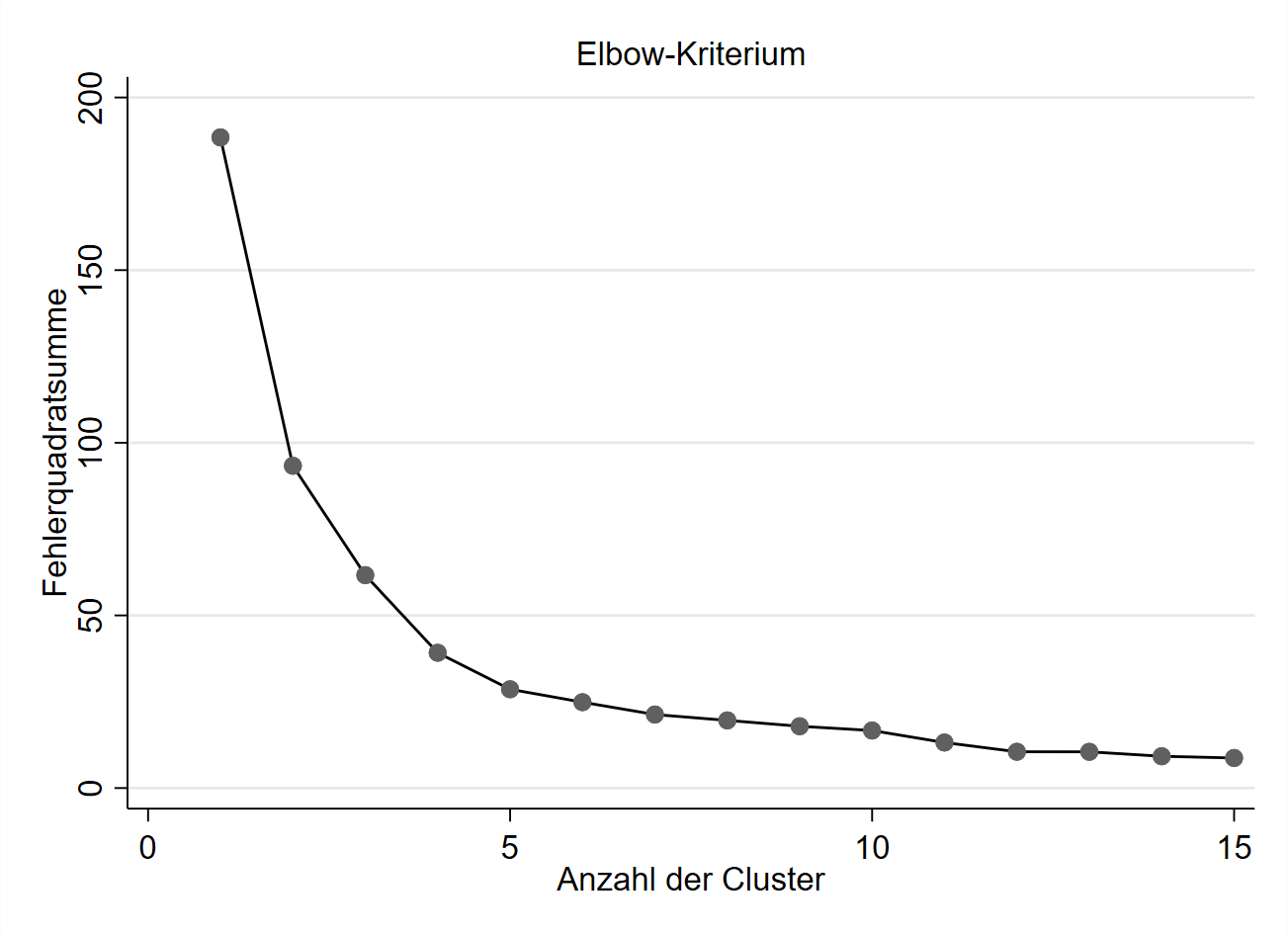


Quelle: SOEP v36, eigene Darstellung

**Online-Anhang**

**Tabelle 1: Variablenbeschreibung**

| Variable | Mittelwert / Anteil | Standard-abweichung | Range | Gültige Werte |
| --- | --- | --- | --- | --- |
| Arbeitsteilung Haushalt (ja/nein): |  |  |  |  |
| *Frau< Partner (Frau übernimmt weniger als 35 Prozent der Tätigkeit)* | 0,02 | 0,14 | 0-1 | n=843 |
| *Frau≈ Partner (Beide übernehmen zwischen 35 und 65 Prozent der Tätigkeit)* | 0,16 | 0,37 | 0-1 | n=843 |
| *Frau> Partner*  *(Frau übernimmt mehr als 65 Prozent der Tätigkeit)* | 0,69 | 0,46 | 0-1 | n=843 |
| Arbeitsteilung Erwerb (ja/nein): |  |  |  |  |
| *Frau<Partner (Frau übernimmt weniger als 35 Prozent der Tätigkeit)* | 0,38 | 0,48 | 0-1 | n=890 |
| *Frau≈Partner (Beide übernehmen zwischen 35 und 65 Prozent der Tätigkeit)* | 0,54 | 0,50 | 0-1 | n=890 |
| *Frau> Partner (Frau übernimmt mehr als 65 Prozent der Tätigkeit)* | 0,08 | 0,28 | 0-1 | n=890 |
| Ostdeutschland (ja/nein) | 0,26 | 0,44 | 0-1 | n=894 |
| Verheiratet (ja/nein) | 0,71 | 0,45 | 0-1 | n=900 |
| Weiteres Kind (ja/nein) | 0,47 | 0,50 | 0-1 | n=900 |
| Alter Frau bei Geburt (in Jahren) | 27,40 | 4,95 | 17-41 | n=900 |
| Alter Partner bei Geburt (in Jahren) | 29,54 | 5,23 | 19-43 | n=900 |
| Bildung Frau (ja/nein): |  |  |  |  |
| *Niedrig (Dauer der Ausbildung: bis 11 Jahre)* | 0,31 | 0,46 | 0-1 | n=864 |
| *Mittel (Dauer der Ausbildung: 11,5 bis 13 Jahre)* | 0,34 | 0,47 | 0-1 | n=864 |
| *Hoch (Dauer der Ausbildung: 13,5 bis 18 Jahre)* | 0,31 | 0,46 | 0-1 | n=864 |
| Bildung Partner (ja/nein): |  |  |  |  |
| *Niedrig (Dauer der Ausbildung: bis 11 Jahre)* | 0,34 | 0,47 | 0-1 | n=878 |
| *Mittel (Dauer der Ausbildung: 11,5 bis 13 Jahre)* | 0,34 | 0,48 | 0-1 | n=878 |
| *Hoch (Dauer der Ausbildung: 13,5 bis 18 Jahre)* | 0,28 | 0,45 | 0-1 | n=878 |

Quelle: SOEP v36, gewichtete Ergebnisse, eigene Darstellung

**Online-Anhang**

**Tabelle 2:** Elementen- und Episodennummer im Kohorten- und Clustervergleich

|  | Elementen-Nummer | Episoden-Nummer |
| --- | --- | --- |
| Kohorte 1970-1974 | 2,17 | 12,31 |
| Kohorte 1975-1979 | 2,19 | 12,36 |
| Kohorte 1980-1984 | 2,54 | 15,38 |
|  |  |  |
| Cluster 1 | 1,58 | 5,32 |
| Cluster 2 | 2,74 | 17,73 |
| Cluster 3 | 2,47 | 16,63 |
| Cluster 4 | 3,00 | 19,21 |
|  |  |  |
| Gesamtlänge (Dauer in Monaten) | 36,00 | 36,00 |

Quelle: SOEP v36, gewichtete Ergebnisse, eigene Darstellung

**Abbildung 2:** Indexplots nach Clustern


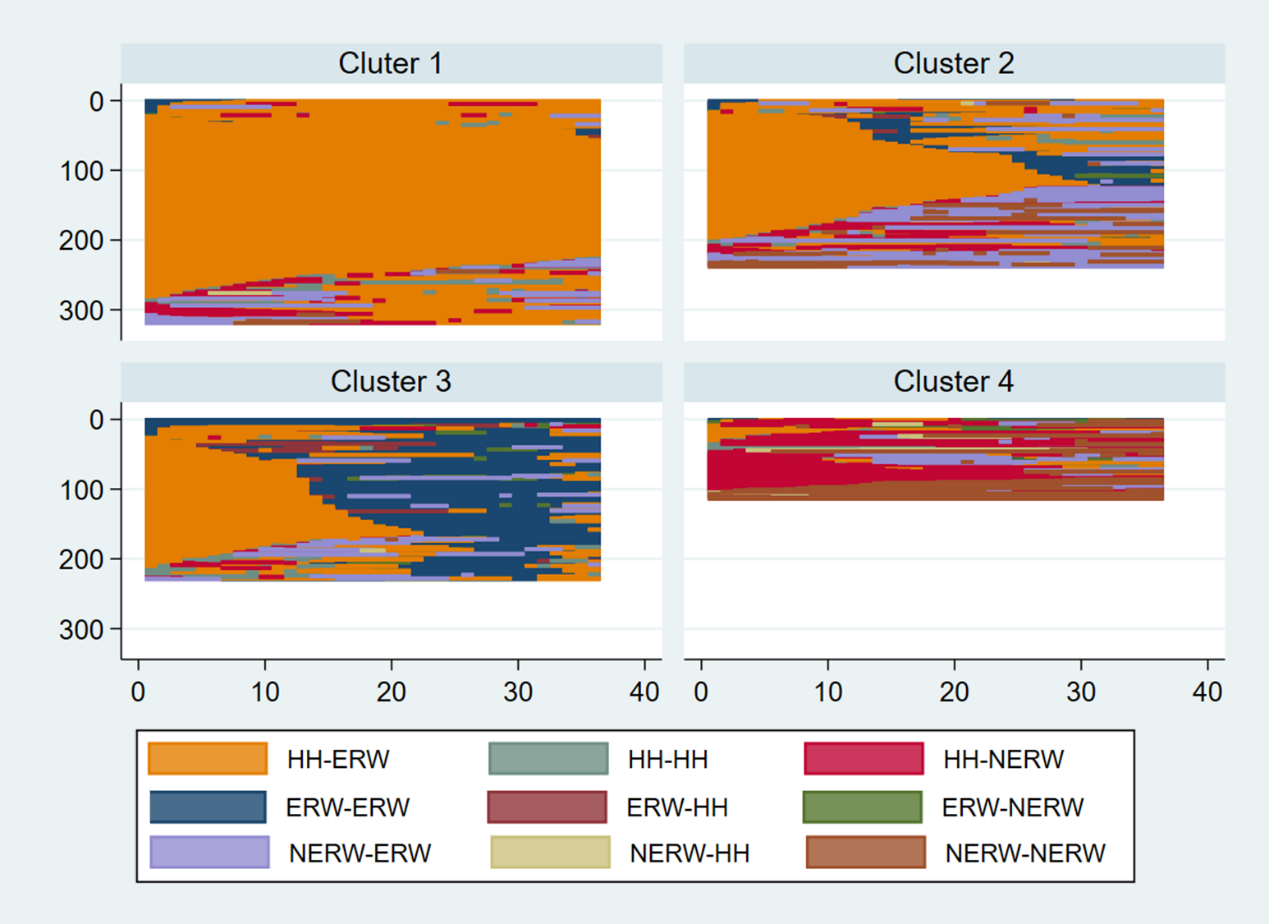


Quelle: SOEP v36, eigene Darstellung

Anmerkung: HH = Haushalt / Elternzeit; ERW = Erwerbstätigkeit; NERW = Nicht-Erwerbstätigkeit;

n(Cluster 1)=319, n(Cluster 2)=238, n(Cluster 3)=229; n(Cluster 4)=114.

**Online-Anhang**

**Tabelle 3:** Ergebnisse der multinomialen logistischen Regression, Darstellung mit Kontrollvariablen (AME)

|  |  | **Cluster 1** | | **Cluster 2** | | **Cluster 3** | | **Cluster 4** | |
| --- | --- | --- | --- | --- | --- | --- | --- | --- | --- |
|  |  | M1.1 | M1.2 | M2.1 | M2.2 | M3.1 | M3.2 | M4.1 | M4.2 |
|  |  | AME *(se)* | AME  *(se)* | AME  *(se)* | AME  *(se)* | AME  *(se)* | AME  *(se)* | AME  *(se)* | AME  *(se)* |
| Kohorte 1975-1979 |  | -0,07+  *(0,04)* | -0,08*  *(0,04)* | 0,04  *(0,04)* | 0,03  *(0,04)* | 0,03  *(0,04)* | 0,04  *(0,04)* | -0,00  *(0,03)* | 0,01  *(0,03)* |
| Kohorte 1980-1984 |  | -0,20**  *(0,04)* | -0,20**  *(0,04)* | 0,05  *(0,04)* | 0,04  *(0,04)* | 0,10**  *(0,04)* | 0,11**  *(0,04)* | 0,05+  *(0,03)* | 0,05  *(0,03)* |
| Arbeitsteilung Haushalt: Frau≈ Partner |  |  | -0,05  *(0,09)* |  | -0,15*  *(0,07)* |  | 0,05  *(0,08)* |  | 0,15  *(0,10)* |
| Arbeitsteilung Haushalt: Frau< Partner |  |  | -0,13***  *(0,04)* |  | -0,02  *(0,04)* |  | 0,00  *(0,03)* |  | 0,15***  *(0,04)* |
| Arbeitsteilung Erwerb: Frau≈Partner |  |  | -0,02  *(0,04)* |  | -0,08*  *(0,04)* |  | 0,10**  *(0,03)* |  | 0,01  *(0,03)* |
| Arbeitsteilung Erwerb: Frau>Partner |  |  | 0,01  *(0,06)* |  | -0,18***  *(0,04)* |  | -0,09+  *(0,05)* |  | 0,25***  *(0,07)* |
| Ostdeutschland |  | -0,21***  *(0,04)* | -0,20***  *(0,04)* | 0,02  *(0,04)* | 0,03  *(0,04)* | 0,13***  *(0,03)* | 0,12***  *(0,03)* | 0,05*  *(0,02)* | 0,06*  *(0,02)* |
| Verheiratet |  | 0,08*  *(0,04)* | 0,07+  *(0,04)* | 0,01  *(0,04)* | 0,00  *(0,04)* | -0,03  *(0,03)* | -0,03  *(0,03)* | -0,07**  *(0,02)* | -0,05*  *(0,02)* |
| Weiteres Kind |  | 0,14***  *(0,03)* | 0,13***  *(0,03)* | 0,01  *(0,03)* | 0,01  *(0,03)* | -0,10***  *(0,03)* | -0,10***  *(0,03)* | -0,04+  *(0,02)* | -0,04+  *(0,02)* |
| Alter Frau |  | -0,01*  *(0,01)* | -0,01*  *(0,01)* | -0,00  *(0,01)* | 0,00  *(0,01)* | 0,02**  *(0,01)* | 0,01**  *(0,01)* | 0,00  *(0,00)* | -0,00  *(0,00)* |
| Pseudo R2 |  | 0,105 | 0,144 | 0,105 | 0,144 | 0,105 | 0,144 | 0,105 | 0,144 |
| *Fallzahl* |  | *900* | *900* | *900* | *900* | *900* | *900* | *900* | *900* |

AME: Durchschnittliche marginale Effekte, *(se)*: Standardfehler **** p<0,001 **p<0,01 *p<0,05 +p<0,10.* Weiterhin wurde auf Alter des Partners sowie auf Bildung (niedrig, mittel, hoch) kontrolliert, nach diesen Merkmalen zeigen sich keine statistisch signifikanten Ergebnisse. Es wurden Kategorien mit fehlenden Werten gebildet und in die Analyse miteinbezogen (in der Tabelle nicht dargestellt); somit bleibt die Fallzahl bei 900 Fällen.

Quelle: SOEP v36, eigene Darstellung
